# Comparison of Preoperative CT Colonography and Colonoscopy for Esophageal Reconstruction with Colonic Interposition

**DOI:** 10.1155/2020/6585762

**Published:** 2020-11-16

**Authors:** Prasit Mahawongkajit, Nuttorn Boochangkool

**Affiliations:** Department of Surgery, Faculty of Medicine, Thammasat University, Bangkok, Pathumthani, Thailand

## Abstract

Colonic evaluation is an essential step before proceeding with esophagectomy to reconstruct by colonic interposition. Colonoscopy is the standard practice for colorectal cancer screening, but it has a chance of failing cecal intubation and carries a risk of horrific adverse events by colonic perforation. CT colonography is a less invasive alternative method reported as useful for colonoscopic screening in cases of average risk of colorectal cancer. This study set out to report our clinical experience and to evaluate CT colonography in the preoperative process for colonic interposition of esophagectomy patients. Data for esophagectomy with colonic interposition patients were retrospectively analyzed and compared the colonoscopy group with the CT colonography group. During eight years, 31 patients, 12 patients in the colonoscopy group and 19 patients in the CT colonography group, included in this study. In both groups, the patient demographic data, procedures, and outcomes were not different. After colonic interposition, endoscopy was performed, and no lesions of conduits were detected. CT colonography is a minimally invasive and reliable option for colonic evaluation method for the patient of average colorectal cancer risk who has undergone esophagectomy with colonic interposition.

## 1. Introduction

Two esophageal diseases, namely, esophageal cancer and corrosive esophageal injury, are significant health problems worldwide, especially in developing countries such as Thailand [1–6]. Esophagectomy is the leading surgical procedure in many accepted cancer treatment guidelines [7–9], for corrosive esophageal stricture and esophageal perforation [10, 11]. Colonic interposition is one of the reconstruction options after esophagectomy for these conditions [12, 13].

Colonic evaluation is an essential step before proceeding with esophagectomy to reconstruct by colonic interposition, particularly in a patient who has a high risk for colorectal cancer or is more than 50 years old. Colonoscopy is the standard practice for colorectal cancer screening by direct visualization of the intraluminal mucosa. However, it has a chance of failing cecal intubation [14, 15] and carries the risk of horrific adverse events by colonic perforation [16]. CT colonography is a less invasive alternative method reported to be useful for colonoscopic screening in cases of average risk of colorectal cancer [17, 18]. This study aimed to report our clinical experience and to evaluate CT colonography in the preoperative process for colonic interposition of esophagectomy patients.

## 2. Patients/Materials and Methods

We conducted a retrospective study with the esophagectomy patients between January 2011 and December 2019, who were identified in our hospital electronic documentation system. This study passed the institutional review board and ethical research process from the Human Ethics Committee of Thammasat University (Faculty of Medicine) with reference number MTU-EC-SU-0-175/62.

In this study, the esophageal cancer patients were treated, and the indication of esophagectomy was made by the gastrointestinal tumor board, which had the treating oncologists, diagnostic radiologists, radiation oncologists, and upper gastrointestinal surgeons as members.

For corrosive injury in our study, the surgical candidate patients consisted of a perforated caustic injury with the previous esophagectomy, and postcorrosive stricture with unsuccessful esophageal dilation. This group of patients obtained nutritional support by feeding jejunostomy and underwent the operation after the corrosive ingestion episode within a period of 6 to 8 months.

The surgical plan for the conduit and reconstruction route was made by the patients and their families with an upper gastrointestinal surgeon. The patients who received a gastric tube, whole stomach, and jejunal graft for reconstruction after esophagectomy and patients with a high risk of colorectal cancer were excluded from this study. The high risk of colorectal cancer can be defined as a history of adenoma or sessile serrated polyps or colorectal cancer; with a history of inflammatory bowel disease; or with a family history of colorectal cancer or confirmed advanced adenomas such as high-grade dysplasia, size >1 cm, villous or tubulovillous histology, or advanced sessile serrated polyps [19]. All of the patients who desired surgery with colonic interposition were assessed by preoperative colonoscopy or CT colonography for colonic evaluation and returned a negative finding of the colon. After surgery, endoscopy was performed for colonic conduit evaluation within three months. The data recorded patient characteristics, surgical procedures, postoperative courses, outcomes, and adverse events with severity by the Clavien–Dindo classification (Table 1) [20, 21]. The collected data were analyzed and presented descriptively using SPSS version 25 (SPSS Inc, Chicago, USA).

## 3. Results

During eight years, Thirty-one patients who were informed of colonic interposition procedures and consented to undergo surgery included in this study with 12 patients in the colonoscopy group and 19 patients in the CT colonography group.

The mean age, gender, and indication were not different between the two groups. The greater part of procedural indications was neoadjuvant, followed by esophagectomy. Additional demographic data are shown in Table 2.

The adverse events and the results by the Clavien–Dindo classification of colonoscopy and CT colonography were not different. The surgical operations and outcomes were not different by the type of conduits, microvascular supercharging, reconstruction routes, and adverse events. The majority of conduits used ileocolonic graft. All of the procedures included anastomosis in the left cervical area. Microvascular supercharging was performed in 25% cases of the colonoscopy group and 47.4% cases of the CT colonography group. Postoperative adverse events were minor leakage, anastomotic stricture, and pneumonia without colonic conduit necrosis, surgical site infection, and 30-day mortality in both groups. After colonic interposition, endoscopic examination detected no lesions of conduits. Additional surgical procedures and outcomes of colonic interposition are shown in Table 3.

## 4. Discussion

Esophagectomy is one of the most invasive surgical procedures in gastrointestinal surgery. The reconstruction operation is challenging for this group of patients. The surgeon has optional organs for reconstruction, such as the stomach, colon, and jejunum. The stomach used as a gastric tube or whole stomach is the most prevalent esophageal reconstruction conduit. However, for corrosive ingestion, some patients also have a gastric injury, especially for patients with a high grade of corrosive damage, and the addition of such an unexpected intraoperative situation, if the gastric conduit is not suitable, makes colon interposition play an essential role.

The colonic interposition could be performed by the left side, right side, and ileocolonic graft as the options for esophagectomy patients [3, 12, 22–24]. Whatever the reconstruction, the quality of the colon should not be disregarded. Preoperative colonic evaluation is the crucial step in providing intraluminal mucosal information for the surgeon who is responsible for performing reconstruction operations upon this group of patients. The literature reports preoperative investigation for colonic interposition being done by colonoscopy and barium enema [12, 23, 24]. CT colonography is a minimally invasive technique for colonic screening while avoiding the perforation risk from the colonoscopy procedure and unsuccessful cecal intubation. The concerns around unexpected perforation from colonoscopy, if it happens in an esophageal cancer patient, are that not only patient suffering is increased but also the operation might also be delayed and might affect the treatment outcome. Also, to complete colonoscopy, especially cecal intubation, it is necessary to gain information for the right side and ileocolonic graft of this operation. Although the colonoscopy group participants did not demonstrate the difference in adverse events, two patients had oxygen desaturation during colonoscopy under intravenous sedation. All patients were treated successfully by oxygen via a nasal catheter with close vital signs monitor. In our study, we applied preoperative CT colonography for the patients who were not at high risk of colorectal cancer, and the results demonstrated it to be safe and effective for assessing the colon before colonic interposition. Endoscopy was performed in all cases after surgery within three months and could not identify mucosal abnormality of conduit that correlated with the result of preoperative CT colonography. For postoperative adverse events, previous studies including a larger number of patients reported anastomosis leakage, 3–35%; conduit necrosis, 0–9%; anastomotic stricture, 6–19%; wound infection, 15.8–21%; pulmonary adverse events, 32.6–37%; and 30-day mortality, 2.1–7.8% [3, 12, 22, 24]. Our study had comparable results of leakage and stricture with fewer pulmonary adverse events. The minor leakage patients were treated conservatively by feeding jejunostomy as nutritional support and closed within two weeks after diagnosis. The leakage patients had become stricture and handled by balloon dilation. All of the patients with pneumonia prescribed an intravenous broad-spectrum antibiotic. Two patients in the colonoscopy group and one in the CT colonography group required respiratory support. We did not find conduit necrosis, surgical site infection, and 30-day mortality in this study. However, any claims to be better or any clear conclusion cannot be made because of the small number of patients.

In this study, CT colonography is a minimally invasive and reliable option for colonic evaluation method for the patient of average colorectal cancer risk who has undergone esophagectomy with colonic interposition. Further large patient number studies with mid- and long-term follow-up, including subsequent endoscopy, are required to assess and confirm this situation. We support preoperative CT colonography as an option in patients who are surgical candidates for esophagectomy and colonic interposition.

## Figures and Tables

**Table 1:** Classification of surgical complications (Clavien–Dindo classification).

Grade	Definition
1	Any deviation from the normal postoperative course without the need for pharmacological treatment or surgical, endoscopic, and radiological interventions
	Allowed therapeutic regimens are drugs as antiemetics, antipyretics, analgetics, diuretics, electrolytes, and physiotherapy. This grade also includes wound infections opened at the bedside.
2	Requiring pharmacological treatment with drugs other than such allowed for grade I complications. Blood transfusions and total parenteral nutrition are also included.
3	Requiring surgical, endoscopic, or radiological intervention
3a	Intervention not under general anesthesia
3b	Intervention under general anesthesia
4	Life-threatening complication (including CNS complications)^*∗*^ requiring IC/ICU-management
4a	Single organ dysfunction (including dialysis)
4b	Multiorgan dysfunction
5	Death of a patient

^*∗*^Brain hemorrhage, ischemic stroke, subarachnoid bleeding, but excluding transient ischemic attacks (TIA); IC: intermediate care; ICU: intensive care unit.

**Table 2:** Characteristics of patients treated by colonic interposition.

	Colonoscopy	CT colonoscopy	*p* value
Procedure, *n*	*n* = 12	*n* = 19	
Age, mean ± SD, years	51.2 ± 15	57.6 ± 13.6	0.24
Sex, male/female	8/3	17/2	0.17
Indication, *n* (%)			
Esophageal cancer			
Neoadjuvant	7 (58.3)	10 (52.6)	0.77
Salvage	2 (16.7)	3 (15.8)	0.95
Corrosive esophageal injury			
Stricture	1 (8.3)	3 (15.8)	0.95
Perforation	2 (16.7)	3 (15.8)	0.77

**Table 3:** Procedures and outcomes of colonic interposition.

	Colonoscopy	CT colonography	*p* value
Procedure, *n*	*n* = 12	*n* = 19	
Adverse events, *n* (%)			
Oxygen desaturation	0 (0)	2 (0)	0.17
Perforation	0 (0)	0 (0)	0
Conduit, *n* (%)			
Left side colon	2 (16.7)	1 (5.3)	0.37
Right side colon	2 (16.7)	2 (10.5)	0.65
Ileocolonic	8 (66.6)	16 (84.2)	0.3
Supercharge, *n* (%)	3 (25)	9 (47.4)	0.21
Route, *n* (%)			
Substernal	8 (66.6)	10 (52.6)	0.45
Subcutaneous	2 (16.7)	9 (47.4)	0.07
Posterior mediastinum	2 (16.7)	0 (0)	0.17
Surgical adverse events, *n* (%)			
Leakage	2 (16.7)	2 (10.5)	0.65
Necrosis	0 (0)	0 (0)	0
Stricture	2 (16.7)	2 (10.5)	0.65
Pneumonia	3 (25)	3 (15.8)	0.56
Surgical site infection	0 (0)	0 (0)	0
Classification of surgical complications (Clavien–Dindo classification), *n* (%)			
Grade 1	2 (16.7)	2 (10.5)	0.65
Grade 2	1 (8.3)	1 (5.3)	0.76
Grade 3			
Grade 3a	2 (16.7)	2 (10.5)	0.65
Grade 3b	0 (0)	0 (0)	0
Grade 4			
Grade 4a	2 (16.7)	1 (5.3)	0.37
Grade 4b	0 (0)	0 (0)	0
Grade 5	0 (0)	0 (0)	0
30-day mortality	0 (0)	0 (0)	0
Detected lesion of colonic conduit from endoscopy	0 (0)	0 (0)	0

## Data Availability

The patient data used to support the findings of this study are restricted by the Human Ethics Committee of Thammasat University (Faculty of Medicine) in order to protect patient privacy. Data are available from Prasit Mahawongkajit (prasit_md@yahoo.com) for researchers who meet the criteria for access to confidential data.
